# Supplementary material for: Computed Tomography Does Not Improve Intra- and Interobserver Agreement of Hertel Radiographic Prognostic Criteria
Source: Medicina (Kaunas). 2022 Oct 19;58(10):1489. doi: 10.3390/medicina58101489 (PMC9612020; doi:10.3390/medicina58101489)
Supplement: Supplementary file 1 [file medicina-58-01489-s001.zip › medicina-1949050-supplementary/Individual folders with instructions on how to respond the survey.pdf]

Individual folders with instructions on how to respond the survey:

- This study seeks to evaluate Hertel's radiographic criteria using computed tomography (CT);
- We separated 20 cases of fractures of the proximal humerus, with X-rays and CT images, individualized in a separate folder;
- Randomization was performed, and the cases were numbered from 1 to 20;
- We ask that each examiner define which Hertel criteria are present by evaluating the radiographs and then their respective CT scans;
- At the end of the evaluation, each examiner will have made 40 evaluations. 2 for each case;
- In the folder with the files there is a photo explaining each of the 3 Hertel criteria, (reproduced from these author original article);
- We kindly ask you to return these results for each patient. Example:
  Case 1: X-ray 1
  --criterion A present/absent/cannot evaluate;
  –criterion B present/absent/cannot evaluate;
  –criterion C present/absent/cannot evaluate;

  CT 1
  --criterion A present/absent/cannot evaluate;
  –criterion B present/absent/cannot evaluate;
  –criterion C present/absent/cannot evaluate;

- The annotation of these results can be done in the way that suits the examiner. Annotated on a sheet of paper, or in an Excell table, or in a .doc text;
- After ending with the document, please send it to the emails: p_tullio@hotmail.com and v_giordano@me.com;
- Very soon we will send the same cases again but with a new randomization for a second evaluation, allowing us to make an intra and interobserver agreement comparison.

Thanks a lot in advance.

Best regards.

Paulo and Vincenzo
