# Supplementary material for: Computed Tomography Does Not Improve Intra- and Interobserver Agreement of Hertel Radiographic Prognostic Criteria
Source: Medicina (Kaunas). 2022 Oct 19;58(10):1489. doi: 10.3390/medicina58101489 (PMC9612020; doi:10.3390/medicina58101489)
Supplement: Supplementary file 1 [file medicina-58-01489-s001.zip › medicina-1949050-supplementary/Supplementary Material Table S1.pdf]

Supplementary Material Table S1. Distribution of criterion A, B, and C by radiographic and CT images between rounds R1 and R2.

Evaluator	Variable	Criteria (A, B, and C)	R1		R2		Agreement (%)	Kappa	p-value
			n	%	n	%			
SHOULDER 1	X-ray C1	P	1	5.0	2	10.0			
		A	9	45.0	6	30.0	70.0	0.46	**0.009**
		I	10	50.0	12	60.0			
	X-ray C2	P	13	65.0	8	40.0			
		A	4	20.0	6	30.0	75.0	0.61	**<0.0001**
		I	3	15.0	6	30.0			
	X-ray C3	P	0	0.0	0	0.0			
		A	15	75.0	17	85.0	70.0	0.08	0.72
		I	5	25.0	3	15.0			
	CT C1	P	3	15.0	5	25.0			
		A	14	70.0	11	55.0	55.0	0.18	0.26
		I	3	15.0	4	20.0			
	CT C2	P	11	55.0	10	50.0			
		A	6	30.0	9	45.0	70.0	0.48	**0.004**
		I	3	15.0	1	5.0			
	CT C3	P	1	5.0	1	5.0			
		A	19	95.0	19	95.0	90.0	"-0.05"	0.81
		I	0	0.0	0	0.0			
SHOULDER 2	X-ray C1	P	6	30.0	8	40.0			
		A	11	55.0	11	55.0	80.0	0.65	**0.0002**
		I	3	15.0	1	5.0			
	X-ray C2	P	9	45.0	10	50.0			
		A	10	50.0	9	45.0	80.0	0.63	**0.001**
		I	1	5.0	1	5.0			
	X-ray C3	P	4	20.0	2	10.0			
		A	11	55.0	14	70.0	65.0	0.36	**0.025**
		I	5	25.0	4	20.0			
	CT C1	P	5	25.0	7	35.0			
		A	15	75.0	13	65.0	80.0	0.53	**0.014**
		I	0	0.0	0	0.0			
	CT C2	P	10	50.0	10	50.0			
		A	10	50.0	10	50.0	80.0	0.60	**0.007**
		I	0	0.0	0	0.0			
	CT C3	P	3	15.0	5	25.0			
		A	17	85.0	15	75.0	80.0	0.38	0.070
		I	0	0.0	0	0.0			
	X-ray C1	P	4	20.0	15	75.0			
		A	11	55.0	4	20.0	35.0	0.11	0.31
		I	5	25.0	1	5.0			
		P	13	65.0	15	75.0			

	X-ray C2	A	7	35.0	5	25.0	80.0	0.53	**0.014**
		I	0	0.0	0	0.0			
		P	1	5.0	2	10.0			
	X-ray C3	A	18	90.0	17	85.0	80.0	0.12	0.47
		I	1	5.0	1	5.0			
		P	11	55.0	13	65.0			
	CT C1	A	9	45.0	7	35.0	80.0	0.59	**0.007**
		I	0	0.0	0	0.0			
		P	13	65.0	18	90.0			
	CT C2	A	7	35.0	2	10.0	75.0	0.34	**0.042**
		I	0	0.0	0	0.0			
		P	1	5.0	1	5.0			
	CT C3	A	19	95.0	19	95.0	100.0	1.0	**<0.0001**
		I	0	0.0	0	0.0			
SHOULDER 3									
		P	2	10.0	18	90.0			
	X-ray C1	A	18	90.0	2	10.0	0.0	“-0.22”	1
		I	0	0.0	0	0.0			
		P	3	15.0	15	75.0			
	X-ray C2	A	17	85.0	5	25.0	10.0	“-0.33”	1
		I	0	0.0	0	0.0			
		P	2	10.0	0	0.0	75.0	n/a	n/a
	X-ray C3	A	15	75.0	20	100			
		I	3	15.0	0	0.0			
		P	2	10.0	18	90.0	0.0	“-0.22”	1
	CT C1	A	18	90.0	2	10.0			
		I	0	0.0	0	0.0			
		P	3	15.0	16	80.0			
SHOULDER 4	CT C2	A	17	85.0	4	20.0	15.0	“-0.20”	1
		I	0	0.0	0	0.0			
		P	3	15.0	4	20.0	75.0	0.14	0.53
	CT C3	A	17	85.0	16	80.0			
		I	0	0.0	0	0.0			
		P	10	50.0	13	65.0			
	X-ray C1	A	7	35.0	5	25.0	65.0	0.39	**0.019**
		I	3	15.0	2	10.0			
		P	15	75.0	15	75.0			
	X-ray C2	A	2	10.0	3	15.0	80.0	0.51	**0.002**
		I	3	15.0	2	10.0			
		P	1	5.0	1	5.0			
	X-ray C3	A	16	80.0	17	85.0	90.0	0.67	**0.0001**
		I	3	15.0	2	10.0			
		P	13	65.0	13	65.0			
	CT C1	A	3	15.0	5	25.0	70.0	0.42	**0.008**
		I	4	20.0	2	10.0			
		P	12	60.0	16	80.0			
	CT C2	A	4	20.0	2	10.0	80.0	0.58	**0.0001**
		I	4	20.0	2	10.0			
		P	2	10.0	1	5.0			
	CT C3	A	14	70.0	17	85.0	85.0	0.61	**0.0001**

TRAUMA 1		I	4	20.0	2	10.0			
	X-ray C1	P	12	60.0	1	5.0			
		A	8	40.0	19	95.0	35.0	"-0.10"	0.21
		I	0	0.0	0	0.0			
	X-ray C2	P	11	55.0	15	75.0			
		A	9	45.0	5	25.0	80.0	0.58	**0.004**
		I	0	0.0	0	0.0			
	X-ray C3	P	4	20.0	5	25.0	85.0	0.57	**0.009**
		A	16	80.0	15	75.0			
		I	0	0.0	0	0.0			
	CT C1	P	12	60.0	1	5.0	35.0	"-0.10"	0.21
		A	8	40.0	19	95.0			
		I	0	0.0	0	0.0			
TRAUMA 2	CT C2	P	11	55.0	15	75.0	80.0	0.58	**0.004**
		A	9	45.0	5	25.0			
		I	0	0.0	0	0.0			
	CT C3	P	3	15.0	7	35.0	80.0	0.49	**0.010**
		A	17	85.0	13	65.0			
		I	0	0.0	0	0.0			
	X-ray C1	P	15	75.0	14	70.0			
		A	3	15.0	6	30.0	80.0	0.53	**0.002**
		I	2	10.0	0	0.0			
	X-ray C2	P	16	80.0	15	75.0			
		A	3	15.0	5	25.0	90.0	0.72	**0.0001**
		I	1	5.0	0	0.0			
	X-ray C3	P	1	5.0	1	5.0			
		A	16	80.0	15	75.0	85.0	0.59	**0.001**
		I	3	15.0	4	20.0			
	CT C1	P	15	75.0	12	60.0			
		A	4	20.0	8	40.0	50.0	"-0.06"	0.73
		I	1	5.0	0	0.0			
	CT C2	P	16	80.0	15	75.0			
TRAUMA 3		A	4	20.0	5	25.0	85.0	0.57	**0.010**
		I	0	0.0	0	0.0			
	CT C3	P	2	10.0	1	5.0			
		A	16	80.0	18	90.0	80.0	0.26	0.10
		I	2	10.0	1	5.0			
	X-ray C1	P	3	15.0	5	25.0			
		A	13	65.0	11	55.0	65.0	0.38	**0.017**
		I	4	20.0	4	20.0			
	X-ray C2	P	13	65.0	13	65.0			
		A	6	30.0	5	25.0	75.0	0.50	**0.006**
		I	1	5.0	2	10.0			
	X-ray C3	P	3	15.0	6	30.0	40.0	0.09	0.53
		A	9	45.0	5	25.0			
		I	8	40.0	9	45.0			
	CT C1	P	7	35.0	10	50.0	85.0	0.70	**0.001**
		A	13	65.0	10	50.0			
		I	0	0.0	0	0.0			
TRAUMA							80.0	0.56	**0.012**

4	CT C2	P	13	65.0	13	65.0	85.0	0.63	**0.004**
		A	7	35.0	7	35.0			
		I	0	0.0	0	0.0			
	CT C3	P	5	25.0	6	30.0			
		A	15	75.0	14	70.0			
		I	0	0.0	0	0.0			

Source: SOT-Nova, HMMC, 2022.
Legends: R1 – round 1; R2 – round 2; % – percentage; n – number of responses; X-ray – radiography; CT – computed tomography; C1 – criterion A; C2 – criterion B; C3 – criterion C; P – present; A – absent; I – inconclusive; n/a – not applicable.
