# Supplementary material for: Computed Tomography Does Not Improve Intra- and Interobserver Agreement of Hertel Radiographic Prognostic Criteria
Source: Medicina (Kaunas). 2022 Oct 19;58(10):1489. doi: 10.3390/medicina58101489 (PMC9612020; doi:10.3390/medicina58101489)
Supplement: Supplementary file 1 [file medicina-58-01489-s001.zip › medicina-1949050-supplementary/Supplementary Material Table S2.pdf]

Supplementary Material Table S2. Interobserver analysis in relation to STANDARD: percentage of cases in agreement with prognostic criteria A, B, and C by radiography and CT of the evaluators in round R1.

Evaluator	Variable	Agreement P (%)	Agreement A (%)	Agreement I (%)	Agreement G (%)	Kappa	p-value
SHOULDER 1	X-ray C1	0.0	55.6	75.0	40.0	0.12	0.38
	X-ray C2	0.68	26.7	100	40.0	0.19	0.06
	X-ray C3	0.0	81.3	33.3	70.0	0.17	0.37
	CT C1	11.1	72.7	n/p	54.0	0.0	0.98
	CT C2	40.0	26.7	n/p	30.0	"-0.10"	0.47
	CT C3	0.0	94.4	n/p	85.0	"-0.07"	0.73
SHOULDER 2	X-ray C1	42.9	66.7	25.0	50.0	0.19	0.24
	X-ray C2	33.3	53.3	0.0	45.0	0.0	0.98
	X-ray C3	100	62.5	33.3	60.0	0.22	0.14
	CT C1	22.2	72.7	n/p	50.0	"-0.05"	0.80
	CT C2	40.0	46.7	n/p	45.0	"-0.10"	0.61
	CT C3	50.0	88.9	n/p	85.0	0.32	0.14
SHOULDER 3	X-ray C1	14.3	55.6	50.0	40.0	0.05	0.74
	X-ray C2	33.3	33.3	0.0	30.0	"-0.09"	0.44
	X-ray C3	100	93.8	0.0	80.0	0.26	0.10
	CT C1	44.4	36.4	n/p	40.0	"-0.19"	0.39
	CT C2	40.0	26.7	n/p	30.0	"-0.22"	0.18
	CT C3	0.0	94.4	n/p	85.0	"-0.07"	0.73
SHOULDER 4	X-ray C1	14.3	88.9	0.0	45.0	0.02	0.87
	X-ray C2	0.0	80.0	0.0	60.0	"-0.18"	0.31
	X-ray C3	0.0	81.3	66.7	75.0	0.33	0.058
	CT C1	22.2	100	n/p	65.0	0.24	0.099
	CT C2	0.0	80.0	n/p	60.0	"-0.23"	0.28
	CT C3	0.0	83.3	n/p	75.0	"-0.14"	0.53
TRAUMA 1	X-ray C1	71.4	44.4	25.0	50.0	0.22	0.18
	X-ray C2	66.7	13.3	0.0	20.0	0.00	0.97
	X-ray C3	0.0	81.3	33.3	70.0	0.10	0.57
	CT C1	66.7	18.2	n/p	40.0	0.04	0.76
	CT C2	60.0	26.7	n/p	35.0	0.07	0.52
	CT C3	50.0	72.2	n/p	70.0	0.17	0.18
TRAUMA 2	X-ray C1	71.4	44.4	0.0	45.0	0.10	0.54
	X-ray C2	33.3	46.7	0.0	40.0	"-0.03"	0.81
	X-ray C3	0.0	81.3	0.0	65.0	0.00	1.00
	CT C1	66.7	45.5	n/p	55.0	0.12	0.58
	CT C2	40.0	40.0	n/p	40.0	"-0.14"	0.44
	CT C3	50.0	88.9	n/p	85.0	0.32	0.14
TRAUMA 3	X-ray C1	85.7	33.3	25.0	50.0	0.23	0.07
	X-ray C2	66.7	20.0	0.0	25.0	0.02	0.84
	X-ray C3	0.0	87.5	66.7	80.0	0.40	**0.029**
	CT C1	78.8	27.3	n/p	50.0	0.10	0.56
	CT C2	80.0	20.0	n/p	35.0	0.00	1.00
	CT C3	0.0	77.8	n/p	70.0	"-0.11"	0.48
TRAUMA 4	X-ray C1	14.3	66.7	75.0	50.0	0.19	0.21
	X-ray C2	33.3	33.3	0.0	30.0	"-0.04"	0.72
	X-ray C3	0.0	56.3	66.7	55.0	0.21	0.12
	CT C1	33.3	63.6	n/p	50.0	"-0.03"	0.89
	CT C2	60.0	33.3	n/p	40.0	"-0.04"	0.79
	CT C3	50.0	77.8	n/p	75.0	0.17	0.39

Source: SOT-Nova, HMMC, 2022.

Legends: R1 – round 1; % – percentage; P – present; A – absent; I – inconclusive; G – general; X-ray – radiography; CT – computed tomography; C1 – criterion A; C2 – criterion B; C3 – criterion C; n/p – not processed.
