# Supplementary material for: Computed Tomography Does Not Improve Intra- and Interobserver Agreement of Hertel Radiographic Prognostic Criteria
Source: Medicina (Kaunas). 2022 Oct 19;58(10):1489. doi: 10.3390/medicina58101489 (PMC9612020; doi:10.3390/medicina58101489)
Supplement: Supplementary file 1 [file medicina-58-01489-s001.zip › medicina-1949050-supplementary/Supplementary Material Table S3.pdf]

Supplementary Material Table S3. Interobserver analysis in relation to STANDARD: percentage of cases in agreement with prognostic criteria A, B, and C by radiography and CT of the evaluators in round R2.

Evaluator	Variable	Agreement P (%)	Agreement A (%)	Agreement I (%)	Agreement G (%)	Kappa	p-value
SHOULDER 1	X-ray C1	0.0	66.7	100	50.0	0.30	**0.019**
	X-ray C2	6.67	40.0	100	50.0	0.27	**0.023**
	X-ray C3	0.0	93.8	66.7	85.0	0.50	**0.011**
	CT C1	33.3	54.5	n/p	45.0	0.06	0.71
	CT C2	40.0	40.0	n/p	40.0	"-0.12"	0.51
	CT C3	0.0	94.4	n/p	85.0	"-0.07"	0.73
SHOULDER 2	X-ray C1	57.1	66.7	0.0	50.0	0.17	0.31
	X-ray C2	33.3	40.0	0.0	35.0	"-0.12"	0.42
	X-ray C3	0.0	81.3	33.3	70.0	0.26	0.13
	CT C1	44.4	72.7	n/p	60.0	0.18	0.42
	CT C2	40.0	46.7	n/p	45.0	"-0.10"	0.61
	CT C3	50.0	77.8	n/p	75.0	0.17	0.39
SHOULDER 3	X-ray C1	85.7	22.2	25.0	45.0	0.14	0.30
	X-ray C2	66.7	26.7	0.0	30.0	0.00	1.00
	X-ray C3	0.0	87.5	0.0	70.0	0.02	0.88
	CT C1	44.4	18.2	n/p	30.0	"-0.36"	0.081
	CT C2	80.0	6.7	n/p	25.0	"-0.07"	0.39
	CT C3	0.0	94.4	n/p	85.0	"-0.07"	0.73
SHOULDER 4	X-ray C1	85.7	11.1	0.0	35.0	"-0.02"	0.87
	X-ray C2	66.7	26.7	0.0	30.0	0.00	1.31
	X-ray C3	0.0	100	0.0	80.0	n/a	n/a
	CT C1	77.8	0.0	n/p	35.0	"-0.20"	0.099
	CT C2	80.0	20.0	n/p	35.0	0.00	1.00
	CT C3	0.0	77.8	n/p	70.0	"-0.15"	0.46
TRAUMA 1	X-ray C1	85.7	44.4	25.0	55.0	0.30	**0.043**
	X-ray C2	66.7	20.0	0.0	25.0	0.02	0.82
	X-ray C3	0.0	87.5	0.0	70.0	0.01	0.96
	CT C1	55.6	18.2	n/p	35.0	"-0.14"	0.40
	CT C2	80.0	13.3	n/p	30.0	0.03	0.69
	CT C3	50.0	88.9	n/p	85.0	0.35	**0.019**
TRAUMA 2	X-ray C1	0.0	88.9	0.0	40.0	"-0.08"	0.30
	X-ray C2	66.7	26.7	0.0	30.0	0.00	1.00
	X-ray C3	0.0	75.0	0.0	70.0	"-0.03"	0.81
	CT C1	0.0	90.9	n/p	50.0	"-0.10"	0.35
	CT C2	80.0	26.7	n/p	40.0	0.04	0.77
	CT C3	50.0	66.7	n/p	65.0	0.08	0.64
TRAUMA 3	X-ray C1	100	55.6	0.0	60.0	0.35	**0.015**
	X-ray C2	66.7	26.7	0.0	30.0	0.00	1.00
	X-ray C3	0.0	81.3	66.7	75.0	0.32	0.083
	CT C1	66.7	45.5	n/p	55.0	0.12	0.58
	CT C2	80.0	26.7	n/p	40.0	0.04	0.77
	CT C3	0.0	88.9	n/p	80.0	"-0.08"	0.64
TRAUMA 4	X-ray C1	57.1	88.9	75.0	75.0	0.60	**0.0001**
	X-ray C2	66.7	26.7	0.0	30.0	0.01	0.95
	X-ray C3	100	31.3	66.7	40.0	0.16	0.10
	CT C1	55.6	54.5	n/p	55.0	0.10	0.65
	CT C2	80.0	40.0	n/p	50.0	0.13	0.42
	CT C3	100	77.8	n/p	80.0	0.41	**0.022**

Source: SOT-Nova, HMMC, 2022.

Legends: R2 – round 2; % – percentage; P – present; A – absent; I – inconclusive; G – general; X-ray – radiography; CT – computed tomography; C1 – criterion A; C2 – criterion B; C3 – criterion C; n/p – not processed; n/a – not applicable.
